# Supplementary material for: NMR-Based Metabolic Profiles of Intact Zebrafish Embryos Exposed to Aflatoxin B1 Recapitulates Hepatotoxicity and Supports Possible Neurotoxicity
Source: Toxins (Basel). 2019 May 8;11(5):258. doi: 10.3390/toxins11050258 (PMC6563017; doi:10.3390/toxins11050258)
Supplement: Supplementary file 1 [file toxins-11-00258-s001.pdf]

Supplementary Materials: NMR-Based Metabolic Profiles of Intact Zebrafish Embryos Exposed to Aflatoxin B1 Recapitulates Hepatotoxicity and Supports Possible Neurotoxicity

Zain Zuberi, Muhamed N. H. Eeza, Joerg Matysik, John P. Berry and A. Alia

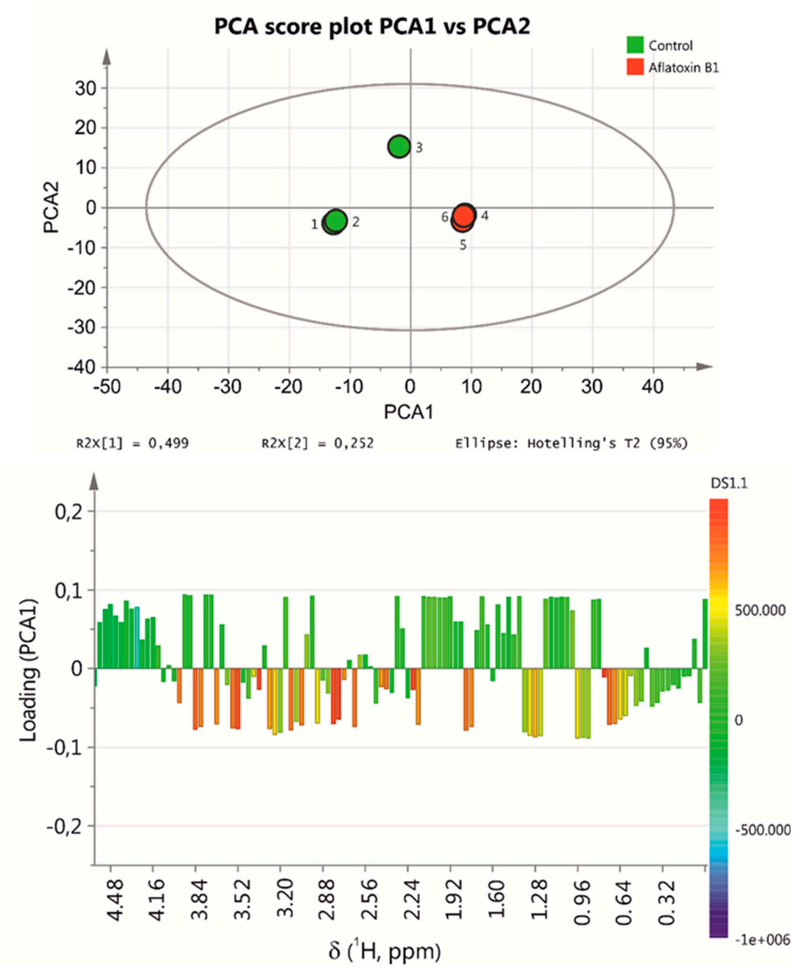

Figure S1. PCA scores plots (**A**) and loading plot (**B**) for control and Aflatoxin B1 treated embryo. Spectra derived from the same group have the same color. Total 75% variables are used to make this score plot.

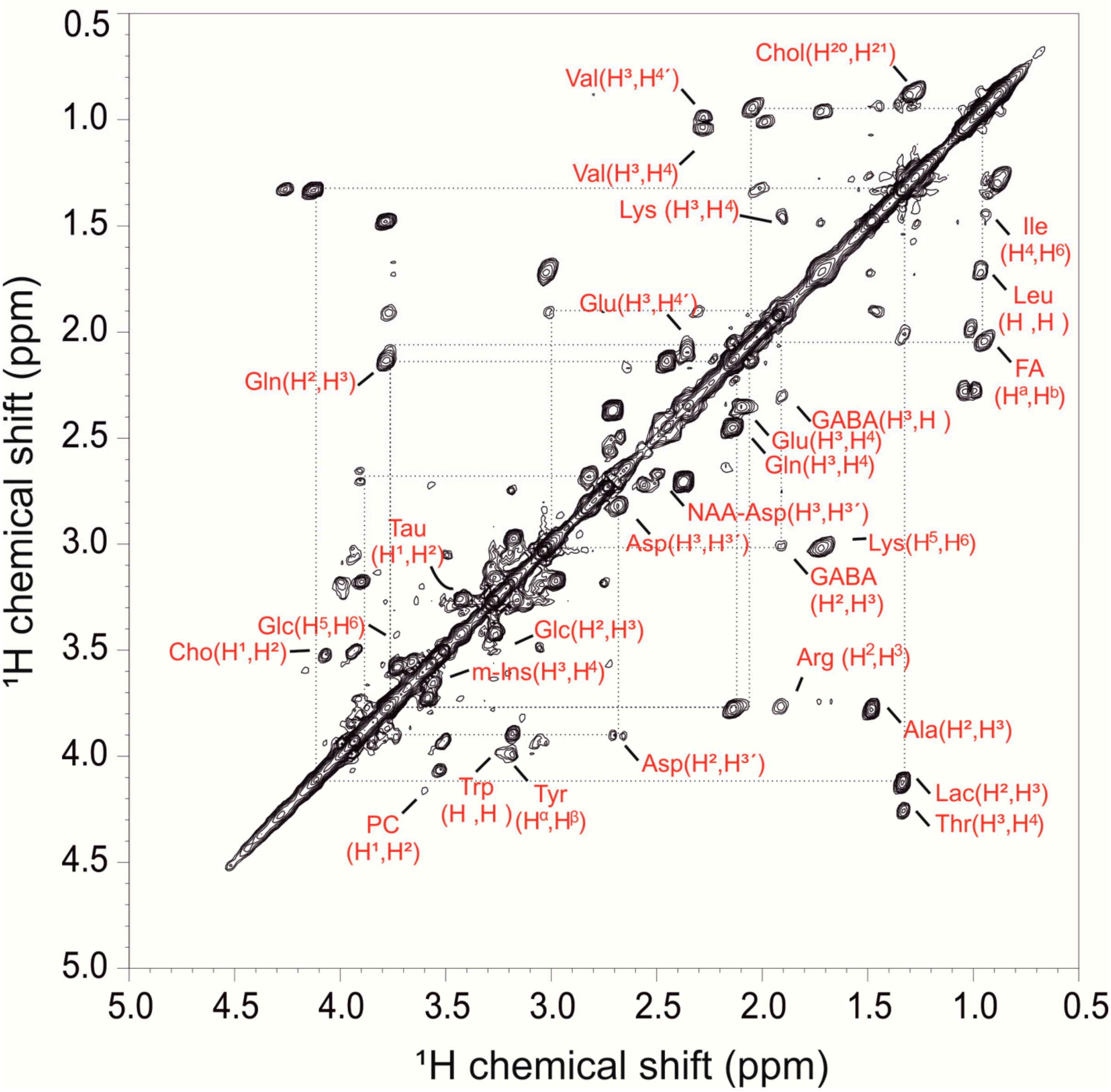

Figure S2. A representative ^{1}H-^{1}H COSY spectra for 96 hpf embryos treated with 1 uM aflatoxin for 24 h. Spectrum was recorded in magnitude mode. The parameters used for COSY were 2048 data points collected in the t2 domain over the spectral width of 4k, 512 t1 increments were collected with 16 transients, relaxation delay 2 sec, acquisition time 116 msec, and pre-saturated water resonance during relaxation delay. The resulting data were zero filled with 512 data points, and were weighted with the squared sine bell window functions in both dimensions prior to Fourier Transformation. Application of gradient pulses along with tradition ^{1}H-^{1}H COSY sequence provides resolution compared to liquid NMR.
